# Clinical Laboratory Validation Study of a High Sensitivity Troponin I Assay on a POCT (Point of Care Testing) Device

**DOI:** 10.5334/gh.1377

**Published:** 2024-12-18

**Authors:** Fabio Grunspun Pitta, Adriana Caschera Leme, Simone Rodrigues Gomes, Tarsila Perez Mota, Fernanda Vieira Paladino, José Leão de Souza Júnior, Rosemeire de Paula Braz, Thais Cristine Rodrigues Leonel Lamounier, Jéssica Buzin Gomes Ferreira, Carlos Eduardo dos Santos Ferreira

**Affiliations:** 1 Hospital Israelita Albert Einstein, Laboratório Clínico, São Paulo – SP, Brazil; 2 Siemens Healthcare Diagnósticos S.A, São Paulo – SP, Brazil

**Keywords:** point-of-care, troponin, chest pain, Acute Coronary Syndrome

## Abstract

**Background:**

In Acute Coronary Syndrome without ST-segment elevation, the use of high-sensitivity troponins in rapid protocols is considered the gold standard for diagnostic exclusion/confirmation, in conjunction with clinical stratification. The biggest concern regarding the techniques for troponin evaluation is the time required between collection and delivery of the result.

**Objective:**

The objective of the present study is the clinical/laboratory validation of a POCT device for TnI.

**Methods:**

In the first phase of the study, samples from 108 patients with known troponin values High Sensitivity Automated Troponin T (TnT) assay from *Roche Diagnostics* were analyzed for analytical comparability between hs-cTnI of the Analyzer Atellica® vTLi and hs-cTnT Cobas®. The second phase of the study was performed with samples from 51 patients who reported to the emergency department with chest pain for a clinical prospective evaluation and correlation between the hs-cTnI assays of the Analyzer Atellica® vTLi, hs-cTnT Cobas® and Atellica IM 1300.

**Results:**

There was a correlation between the POCT Atellica® vTLi and hs-cTnT Cobas® in the serum samples of the control group (r = 0.660, p < 0.0001). Besides, there was a correlation between the Atellica® vTLi, serum hs-cTnT Cobas®, plasma hs-cTnT Cobas®, serum Atellica IM and plasma Atellica IM 1300 platforms in the second phase (p < 0.0001 in all cases).

**Conclusion:**

In the present study, the Siemens POCT Atellica® vTLi device showed excellent performance in laboratory validation and correlation with the high-sensitivity TnT assay in different troponin concentration ranges. Given these results, the device can be used in institutions that intend to use a POCT device for 0- and 1-hour chest pain protocols.

## Introduction

Cardiovascular disease (CVD) is the leading cause of death in the world, causing the death of approximately 17.9 million people in 2019 (1). In 2019, 117,549 deaths were registered in Brazil caused by ischemic heart disease (2).

Among CVDs, myocardial infarction has a high prevalence, high morbidity, and mortality rates (3). The clinical syndrome that characterizes infarction is acute coronary syndrome (ACS), which can be categorized as having or not having ST-segment elevation on the electrocardiogram (4). The diagnosis of ST-segment elevation ACS is easily confirmed by ECG, and biomarkers in blood are used only for prognostic purposes. ACS without ST-segment elevation is the most common clinical syndrome (75–85%), has a high mortality rate in the first hour, and requires the use of high-sensitivity troponins (5).

In ACS without ST-segment elevation, the use of high-sensitivity troponins used in rapid protocols (0/1 h) is considered the gold standard for diagnostic exclusion/confirmation, in conjunction with clinical stratification (6).

Cardiac troponin (cTn) in its two distinct subunits (cTnI and cTnT) has been used as a biomarker of acute myocardial infarction, especially in cases of non-ST-segment elevation infarction (NSTEMI), where diagnosis by electrocardiogram becomes more complex due to the difficulty in identifying the pathological waves in these cases (7,8,9,10,11). Troponins are the biochemical markers of choice with grade A class I recommendation (12,13).

In the laboratory context, high-sensitivity troponins are performed by techniques derived from the Ag-Ac interaction (Chemiluminescence, Electrochemoluminescence, and derivatives). Troponins I and T (TnI and TnT) present high specificity for cardiac muscle and are available for clinical practice on different platforms (12,13).

In general, the most used laboratory methods for measuring troponin levels are based on chemiluminescence and electrochemiluminescence. The biggest concern regarding these techniques is the time required between collection and delivery of the result. This response time (TAT) is usually 1 hour for the availability of results and consequent clinical follow-up (14,15). This is a critical point in the management of patients with coronary disease and acute myocardial infarction since there is evidence that the shorter the time for diagnosis and treatment, the lower the rates of complications, morbidity, and mortality (16,17), showing the importance of developing more practical and faster techniques for this diagnosis.

A possible solution is the development of devices that measure the troponin level with rapid availability of results, with POCT (point of care testing) platforms and analytical sensitivity that allow the implementation of care protocols for chest pain that enable a quick decision making – two dosages 0 and 1 h (18). POCT devices should be portable, practical, and usable at emergency triage stations, in the doctor’s office, or wherever is convenient (19,20).

The development and validation of POCT devices with the objective of evaluating patients with suspected myocardial infarction is still very important, since they can measure patients’ troponin levels quickly, accurately, and in any location–saving a lot of time, which is precious for patients and their clinical outcomes (21).

Despite this relevance, there are still not many POCT devices on the market that can perform a reliable diagnosis based on troponin levels with high sensitivity that allows them to be used in rapid protocols (22).

In this sense, the objective of the present study is the clinical/laboratory validation of a POCT device for TnI, which is based on an immunoassay for the quantification of troponin levels in patients with suspected myocardial infarction. Because it is portable, lightweight, requires a small sample volume (from 30 to 100 μL), has low complexity in sample processing, and provides results in a short period of time (maximum 8 minutes), this is an interesting device for testing at the bedside for patients with suspected acute myocardial infarction.

## Materials and Methods

### Study design and recruitment of patients

This clinical laboratory validation study of the high-sensitivity troponin I assay on a POCT device was carried out in a 750-bed hospital complex. It was conducted by the Cardiology Department, Emergency Department, and Department of Laboratory Medicine. The hospital is accredited by the Joint Commission and the Clinical Laboratory is accredited by the College of American Pathologists (CAP).

The TnT levels of these samples, both serum and plasma, were quantitatively evaluated using electrochemiluminescence methodology, already used by the hospital in an automated instrument from *Roche Diagnostics*. TnI was analyzed on the POCT device Atellica® vTLi, developed by Siemens Healthineers, using chemiluminescence methodology in whole blood samples.

The analytical performance reported by the manufacturers in the reagent label are presented in the Supplementary Table 1.

**Figure 1:**
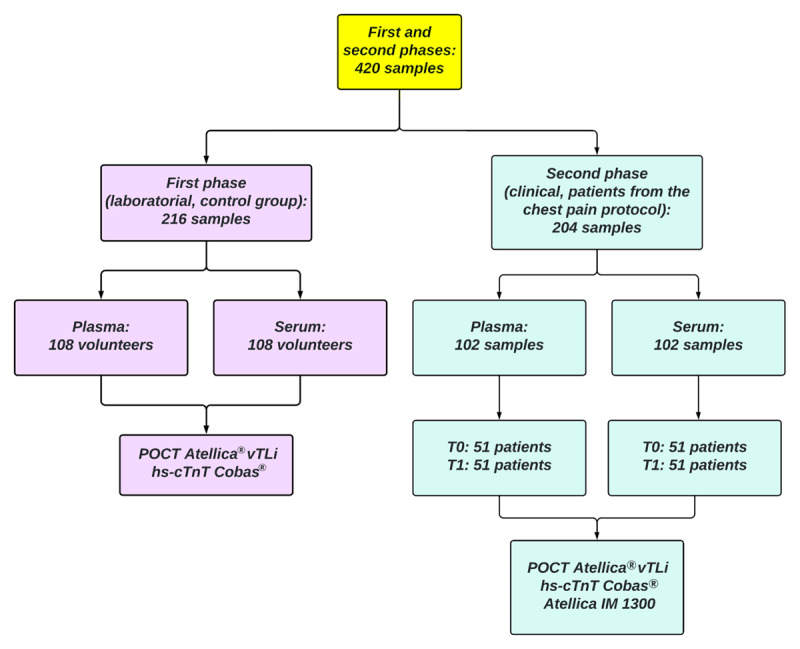
Flow chart of the study.

The study was carried out in two stages (Figure 1), the first involving validation of the device in the Clinical Laboratory and the second corresponding to the routine clinical and laboratory evaluation of patients included in the chest pain protocol. The laboratory stratification of our protocol was based on the 2018 publication for rapid protocols (23).

In the first phase (laboratory validation), it was processed the comparison of samples from 108 patients with known troponin values High Sensitivity Automated Troponin T (TnT) assay from *Roche Diagnostics*. This TnT assay is used routinely in the hospital. We analyzed 77 troponin samples below 52 ng/L and 31 samples with values above 52 ng/L (both serum and plasma), with the aim to evaluate the laboratory performance of the high sensitivity troponin I assay (TnI) from POCT Atellica® vTLi device from Siemens Helthnieers. In addition to this laboratory validation phase, two samples with known concentrations of troponin (internal quality controls), with low and high concentrations, were processed and analyzed four times a day for five days to calculate the coefficient of variation (CV) and its standard deviation (SD), totaling 40 samples over the five days.

The second phase of the study included 51 consecutive patients who were included in the Institution’s chest pain protocol. This group of patients followed the institutional chest pain protocol–TnT measurement. Plasma and serum samples were collected for TnI processing on the POCT Atellica® vTLi, hs-cTnT Cobas®, and Atellica IM 1300. Collections were standardized according to the Clinical Laboratory Standard Institute (CLSI) technical procedures and two tubes were obtained –one tube of whole blood with anticoagulant (lytic heparin) to obtain plasma and a second tube without anticoagulant to obtain serum.

In the clinical stratification of patients from phase 2, the hospital uses the HEART score (24).

In the entire validation study (laboratory–first phase: n = 108 patients (plasma and serum); clinical–second phase: n = 51 patients, plasma and serum in two times = 204), 420 paired samples were processed using our reference methodology (TnT) at the hospital routine of patients treated through the chest pain protocol with the high-sensitivity TnI assay of the POCT Atellica® vTLi (Figure 1).

In addition, we used our chest pain protocol to evaluate the trials, and the *Rule in and Rule out* laboratory criteria, based on the previous published protocol of stratification (23).

***Rule in:*** Troponin values greater than 52 ng/L, or variations between time 0 h and 1 h greater than 3 ng/L when the initial troponin (time 0) is between 5 and 12 ng/L, or variations between time 0 h and 1 h greater than 5 ng/L when the initial troponin (time 0) is between 12 and 52 ng/L.

***Rule out:*** Values below 5 ng/L or variations between time 0 h and 1 h below 3 ng/L when the initial troponin (time 0) is between 5 and 12 ng/L, or variations between time 0 h and 1 h are lower than 5 ng/L when the initial troponin (time 0) is between 12 and 52 ng/L.

## Patient inclusion and exclusion criteria

For the laboratory (first phase), samples from patients with normal troponin values and samples with elevated troponins were selected in our laboratory.

For the clinical laboratory evaluation (second phase), samples were evaluated from patients over 18 years of age who were admitted to the Hospital Emergency Department with suspected myocardial infarction and who were included in the chest pain protocol. All patients who did not sign the form were excluded from the study.

## Ethical Aspects

The study was approved by the Ethics Committee from Hospital Israelita Albert Einstein and followed the principles from the Helsinki Declaration. Patients signed an informed consent.

## Statistical analysis

The SPSS program version 25.0 was used and a significance level of p < 0.05 was considered.

Data were initially evaluated to verify their normality by using the Shapiro–Wilk test and the homogeneity of variance was assessed by the Levene test. Parametric variables were presented as mean and standard deviation (X ± SD). Pearson’s rank correlation coefficient analysis was used to evaluate the association between the assays both in the first and second phases of the study.

## Results

Table 1 shows the values concerning the Quality Control (QC). For QC1 (13.0–29.1 ng/L), the average evaluation range (SD) was 17.05 (1.28), and CV% = 7.5. For QC2 (22.6–51.5 ng/L), the evaluation range was 28.48 (2.47), and CV% = 8.68. For QC3 (238–499 ng/L), the evaluation range was 284.08 (18.90) and CV% = 6.65.

**Table 1:** Quality Control–POCT Atellica® vTLi.


LEVEL 1 (13.0–29.1 ng/L)					

ng/L	First	Second	Third	Fourth	Fifth

Rep 1	18.2	19.0	17.6	19.6	19.9

Rep 2	17.7	16.8	14.2	17.5	16.0

Rep 3	16.3	17.2	15.6	16.8	15.5

Rep 4	17.7	17.3	16.9	16.8	16.

Rep 5	16.9	17.0	15.6	17.9	15.6

**LEVEL 2 (22.6–51.5 ng/L)**					

ng/L	First	Second	Third	Fourth	Fifth

Rep 1	30.5	30.4	32.2	26.7	31.3

Rep 2	28.8	26.5	23.5	29.0	27.3

Rep 3	32.6	31.2	29.1	29.0	22.8

Rep 4	30.3	26.9	30.1	28.4	27.9

Rep 5	30.2	28.2	24.2	27.3	27.6

**LEVEL 3 (238–499 ng/L)**					

ng/L	First	Second	Third	Fourth	Fifth

Rep 1	288	264	258	335	284

Rep 2	291	290	307	268	264

Rep 3	290	252	289	305	288

Rep 4	315	292	267	277	284

Rep 5	292	291	256	284	271


CV (Coefficient of variation): CV Level 1 = 7.5%; CV Level 2 = 8.7%; CV Level 3 = 6.7%. Rep: replicate.

Besides, we found significant correlations between the POCT Atellica® vTLi (plasma samples) and hs-cTnT Cobas® in the serum samples of the control group (in the first phase), and in the first phase (Pearson’s correlation, Table 2).

**Table 2:** Correlations between the assays, group of control samples (first phase).


ASSAYS	R	P

Plasma POCT – VLTi a × Serum Cobas, troponin <5.0	0.462	0.009

Plasma POCT – VLTia × Serum Cobas, troponin >5.0	0.645	<0.0001

Plasma POCT – VLTi a × Serum Cobas, troponin <52.0	0.526	<0.0001

Plasma POCT – VLTia × Serum Cobas, troponin >52.0	0.558	0.001


r: Pearson correlation.

Table 3 presents the clinical data of the patients of the second phase. The mean age (standard deviation) was 61 (16), and 76% were male. Concerning cardiovascular risk factors, 60% presented Systemic Arterial Hypertension, 35% had Diabetes Mellitus, 53% had Dyslipidemia, 100% did not smoke, and 10% had a previous Acute Myocardial Infarction.

**Table 3:** Clinical parameters from the patients with chest pain, second phase of the study.


VARIABLE	VALUES

Age, mean (SD)	61 (16)

Sex, male (%)	76

Systemic Arterial Hypertension (%)	60

Diabetes Mellitus (%)	35

Dyslipidemia (%)	53

Smoking (%)	0

Previous Acute Myocardial Infarction (%)	10

Heart Score 1 point (n)	6

Heart Score 2 points (n)	7

Heart Score 3 points (n)	11

Heart Score 4 points (n)	9

Heart Score 5 points (n)	8

Heart Score 6 points (n)	4

Heart Score 7 points (n)	2

Heart Score 8 points (n)	3

Heart Score 9 points (n)	1


Table 4 represents the Rule out from the patients of phase 2. The similarity between the two methods in evaluating laboratory stratification was 98%. Of the 51 patients included in the second phase, 45 were ruled out, 5 were ruled in and 1 was discordant.

**Table 4:** *Rule in and Rule out* from the patients of phase 2.


PATIENT	POCT VLTia T0	POCT VLTia T1	Plasma COBAS T0	Plasma COBAS T1	RULE IN	RULE OUT

1	7	8	5	4		X–Y

2	16	17	5	5		X–Y

3	37	34	41	38		X–Y

4	3	3	8	8		X–Y

5	6	10	4	4		X–Y

6	2	4	3	3		X–Y

7	4	5	5	5		X–Y

8	9	9	14	16		X–Y

9	6	4	8	5		X–Y

10	7	10	34	32		X–Y

11	7	6	6	5		X–Y

12	3	4	13	15		X–Y

13*	35	49	36	35	X	Y

14	13	14	9	9		X–Y

15	8	9	27	28		X–Y

16	3	3	28	33		X–Y

17	47	43	12	12		X–Y

18	4	5	6	6		X–Y

19	2	1	12	11		X–Y

20	4	5	3	4		X–Y

21	11	10	7	7		X–Y

22	5	7	3	4		X–Y

23	5	5	23	26		X–Y

24	48	177	40	123	X–Y	

25	1250	1250	249	275	X–Y	

26	4	5	13	12		X–Y

27	6	6	7	7		X–Y

28	13	11	14	12		X–Y

29	6	5	19	17		X–Y

30	6	3	6	7		X–Y

31	15	14	45	44		X–Y

32	12	81	49	67	X–Y	

33	5	9	9	14	X–Y	

34	100	462	86	185	X- Y	

35	11	9	3	3		X–Y

36	49	52	45	38		X–Y

37	15	15	15	15		X–Y

38	3	3	5	6		X–Y

39	7	6	7	6		X–Y

40	10	8	22	21		X–Y

41	4	6	4	4		X–Y

42	14	14	19	18		X–Y

43	3	5	8	7		X–Y

44	5	6	13	13		X–Y

45	6	5	8	9		X–Y

46	1	1	8	9		X–Y

47	7	9	10	10		X–Y

48	6	5	22	22		X–Y

49	4	5	7	8		X–Y

50	5	4	3	3		X–Y

51	4	3	10	9		X–Y


*Patient 13: discordant; X: laboratorial stratification of POCT VLTia; Y: laboratorial stratification of Plasma COBAS, according to the chest pain protocol.

Table 5 shows a correlation between the Atellica® vTLi, serum hs-cTnT Cobas®, plasma hs-cTnT Cobas®, serum Atellica IM, and plasma Atellica IM 1300 platforms in the second phase (p < 0.0001 in all cases. Pearson’s correlation), considering the reference values of each manufacturer and the biochemical differences of the troponin I and troponin T molecules.

**Table 5:** Correlations between the assays, second phase of the study (clinical).


ASSAYS	r T0	p T0	r T1	p T1

Whole blood POCT Atellica® vTLi × plasma hs-cTnT Cobas®	0.926	<0.0001	0.927	<0.0001

Whole blood POCT Atellica® vTLi × Serum Cobas	0.956	<0.0001	0.957	<0.0001

Whole blood Plasma Cobas × Serum Cobas	0.995	<0.0001	0.994	<0.0001

Whole blood POCT Atellica® vTLi × Plasma Atellica IM 1300	0.964	<0.0001	1.000	<0.0001

Whole blood POCT Atellica® vTLi × Serum Atellica IM 1300	0.999	<0.0001	0.982	<0.0001

Plasma Atellica IM 1300 × Serum Atellica IM 1300	0.926	<0.0001	1.000	<0.0001


T0: until 3 h since the beginning of the symptoms; T1: Time 1: after 1 h since the symptoms have been detected.

## Discussion

The use of POCT devices in medical practice has increased in recent years and should be expanded given the expansion and improvement of the methodologies used, allowing an increase in the tests offered and an improvement in analytical performance (18).

However, an important point is the safety and quality of using a device by carrying out a validation process–which did in this study.

The assays must have at least a functional sensitivity of 5 ng/L (or pg/mL) or less so that they can be used in clinical practice in rapid protocols. These levels of functional sensitivity are widely available in large, automated platforms described above. They are inserted within a medium and large laboratory context, which limits the use of high-sensitivity Tn in health services that do not have this central laboratory structure or are at a distance that makes rapid diagnosis unfeasible.

The validation of this study aims to evaluate the performance of high-sensitivity troponin TnI on a POCT/VLTI device. In the first phase, we evaluated the analytical performance of the device against a positive control and in a direct comparison with previously selected samples with known high-sensitivity TnT values used in hospital routine. The assessment of analytical performance was based on our clinical laboratory’s method validation protocol. This validation does not aim to analyze the information contained in the package inserts of diagnostic kits (blank limit, sample stability, and analytical and functional sensitivities) (25,26).

For the troponin data obtained in validation (phase 1), samples were selected from different value ranges (<5.0 ng/L, >5.0 ng/L, <52.0 ng/L, and >52.0 ng/L). Since they were different trials, there were differences between the means obtained, despite the good correlation between them (Table 2). Given this, a point to be discussed is that there are dissimilarities between the different troponin measurement methods–and depending on the assay, there may be different basic results (input) of the patients, evidenced both in the laboratory part of our study and in phase 2. These variations occur because each manufacturer developed their kit based on an epitope of the troponin molecule and the reactions are based on Antigen-Antibody (Ag-Ab) interactions that can undergo variations/interference (heterophilic antibodies or macro troponins). As long as there is no harmonization between the different tests, differences will continue to exist (27,28,29,30).

In the clinical assessment of the patient in rapid chest pain protocols, it is essential to use a cutoff of 5 ng/L to exclude cases of infarction (100% negative predictive value) in patients within 3 hours of the onset of symptoms. In addition, to increase the positive predictive value, it is important to check the increase in troponin concentration in the second collection (1 h) (23,30). With the evaluation carried out, it was possible to identify the excellent performance of the POCT device compared to the automated TnT assay.

In phase 2, there were differences between the mean baseline values of the different trials, as already described in phase 1 of the present study. However, both trials showed excellent similarity in the laboratory stratification of the chest pain protocol (Table 4).

The similarity between the two methods in evaluating laboratory stratification was 98%. Of the 51 patients included in the second phase, 44 were rule out, 6 were rule in, and 1 was discordant (Table 4). The only discrepancy was in a patient who already had high troponin values in both methods at time zero and at time 1 h, the values reached more than 5 ng/L in POCT (t0–35 ng/L/t1–49 ng/L) compared with the method used in the routine (t0–35 ng/L/t1–35 ng/L). Having no impact on patient safety as it would be another Rule in if we considered the POCT trial and the patient was not discharged from the hospital as he was a high cardiovascular risk patient in the clinical stratification.

The use of these bedside POCT devices can be used in physician offices, emergencies, inpatient units, pharmacies, surgical centers, intensive care units, and means of transport–and depends on specific legislation of each country. Another important point to highlight in these devices is the operator. Depending on the health service and local legislation, they can be used by doctors, nurses, technicians, and laboratory analysts. These professionals must be trained periodically, and reports must be generated with all the information necessary for their correct interpretation and thus guarantee patient safety. Just like automated laboratory equipment, POCT devices must follow rules for using internal and external quality controls (31).

With the recent approvals by regulatory agencies for POCT devices, clinical studies are beginning to be published with the aim of identifying cutoff points for rapid protocols aimed at safely discharging patients early. In another study (32) using the same device as ours, a value of 4 pg/mL to rule out the diagnosis in a first measurement and a value below 6 pg/mL with a variation of less than 5 pg/mL in a second measurement after 2 h were used. For inclusion of patients, values above 60 pg/mL or variations above 15 pg/mL were used. The exclusion values used in our study were very close. Since we performed the head-to-head comparison with our chest pain protocol (0/1 h), we do not have samples at 2 hours to perform an evaluation with the cutoff points suggested by this study (32).

Finally, to release the results, the automated test takes an average of around 1 hour on the entire process (from request to report release). The test carried out on a POCT device takes around 20 minutes for the entire process (33). However, to ensure the safety of this process, it is important to try to enable all connectivity: insertion of patient data into a laboratory information system (LIS), sample registration, barcode label generation, and release of an interfaced result without the need to type the result or directly view it on the POCT device (34). Another advantage is that because individualized cartridges are used, a more cost-effective practice is possible for services with low demand since the presentation of automated kits is approximately 100 tests per kit.

## Conclusion

In the present study, the Siemens Atellica® vTLi POCT device showed excellent performance in laboratory validation, as well as a correlation and high similarity with the high-sensitivity TnT assay in different troponin concentration ranges, compared to the institutional rapid chest pain protocol (0 and 1 hour). To better validate this POCT device using the proposed cutoff points, studies with clinical follow-up of patients will be necessary.

**Supplementary Table 1:** Analytical specifications reported by the manufacturers.


	TnI – ATELLICA vTLi – PLASMA	TnI – ATELLICA vTLi – TOTAL BLOOD	TnT – COBAS 602 – SERUM

Blanc limit (LoB)	0.55 ng/L	0.55 ng/L	2.36 ng/L

Detection limit (LoD)	1.2 ng/L	1.6 ng/L	2.85 ng/L

Quantification limit (LdQ)	2.1 ng/L	3.7 ng/L	2.92 ng/L

Overall Percentil 99	22.9 ng/L	22.9 ng/L	14 ng/L

